# Youth with Avoidant/Restrictive Food Intake Disorder: Examining Differences by Age, Weight Status, and Symptom Duration

**DOI:** 10.3390/nu11081955

**Published:** 2019-08-20

**Authors:** Kristina Duncombe Lowe, Timothy L. Barnes, Carolyn Martell, Helene Keery, Sarah Eckhardt, Carol B. Peterson, Julie Lesser, Daniel Le Grange

**Affiliations:** 1 Center for the Treatment of Eating Disorders, Children′s Minnesota, Minneapolis, MN 55404, USA; 2 Children’s Minnesota Research Institute, Children′s Minnesota, Minneapolis, MN 55404, USA; 3 Department of Psychiatry, University of Minnesota, Minneapolis, MN 55454, USA; 4 Rogers Behavioral Health, Eden Prairie, MN 55344, USA; 5 Department of Psychiatry and Department of Pediatrics, University of California, San Francisco, CA 94143, USA; 6 Department of Psychiatry and Behavioral Neuroscience, The University of Chicago, Chicago, IL 60637, USA

**Keywords:** avoidant/restrictive food intake disorder, age, weight status, symptom duration, lack of interest, fear of aversive consequences, sensory sensitivity

## Abstract

The primary purpose of this study was to examine differences among youth with avoidant/restrictive food intake disorder (ARFID) by age, weight status, and symptom duration. A secondary goal was to report the frequencies of ARFID using DSM-5 clinical presentations (i.e., fear of aversive consequences, lack of interest in food, sensory sensitivities). Participants (N = 102), ages 8–18 years, were recruited through an eating disorder service within a pediatric hospital. They were evaluated using semi-structured interviews and questionnaires. Patients were assigned to groups according to age, weight status, and symptom duration. Frequencies of clinical presentations, including combinations of DSM-5 categories, were also examined. Our findings suggest that adolescents presented with higher rates of Depression (*p* = 0.04). Youth with chronic ARFID symptoms presented with significantly lower weight (*p* = 0.03), and those with acute symptoms rated significantly higher suicidal ideation and/or self- harm (*p* = 0.02). Half of patients met criteria for more than one ARFID symptom presentation. This study provides preliminary evidence that youth with ARFID differ in clinical presentation depending on age, weight status, and symptom duration, and highlights safety concerns for those with acute symptoms of ARFID. High rates of overlapping symptom presentations might suggest a dimensional approach in the conceptualization of ARFID.

## 1. Introduction

Avoidant/restrictive food intake disorder (ARFID) is a recently recognized eating disorder that was introduced in the Diagnostic and Statistical Manual of Mental Disorders—Fifth Edition (DSM-5) [1]. ARFID is characterized by weight loss or low BMI, nutritional deficiency, medical complications, and/or impaired psychosocial functioning due to eating difficulties [2,3,4]. Current research highlights the variability of clinical presentations within the disorder [2,5]. While research is exploring strategies on how best to describe the heterogeneity within ARFID, the DSM-5 proposes three presentations to guide clinicians in the diagnosis and treatment of ARFID: lack of interest in food, avoidance of food due to aversive sensory characteristics, and concern over negative outcomes of eating (e.g., vomiting, choking) [1].

There is a growing literature examining the clinical differences between individuals presenting with ARFID in comparison to other eating disorders (e.g., anorexia nervosa [AN], bulimia nervosa [BN]). Findings suggest that youth with ARFID are significantly more likely to develop this disorder at a younger age, experience a longer duration of symptoms, are more likely to be male, have longer inpatient stays for medical stabilization, and present with weights higher than those with AN but lower than those with BN [2,3,4,6,7]. Previous research in the eating disorder literature suggests that clinical and medical presentations differ depending on eating disorder diagnosis, age, weight status, and symptom duration and frequency [6,8,9,10]. Given that ARFID is a recent addition to the DSM-5, research efforts to describe the heterogeneity within ARFID are still underway [2,11,12,13,14]. Research in this area is difficult due to the complexities inherent to ARFID, including lack of consensus over clear categorization methods, small sample sizes, and overlapping clinical presentations. Studies have looked at group differences between DSM-5-designated ARFID presentations across clinical characteristics, including psychiatric and medical comorbidities, as well as treatment trajectories [6,12,14]; however, there are limitations to the generalizability of findings due to small sample sizes. Given the current state of the literature, the present study aimed to explore group differences within ARFID defined by age, weight status, and symptom duration, as these variables have been identified as significant correlates for other eating disorders, although their impact within ARFID remains poorly understood [6,9]. Our exploratory study aims to provide groundwork for future hypothesis testing studies in order to better understand the heterogeneity of ARFID. This study utilizes data from a large sample of youth, who were diagnosed with ARFID at the time of intake (rather than via retrospective chart review). Additionally, our study aimed to advance the literature by describing the frequency of clinical presentations using the DSM-5 categories (i.e., sensory sensitivity, fear of aversive consequences, and lack of interest in eating), including combinations of these features.

## 2. Materials and Methods

Participants. Youth aged 8–18 years old (N = 102) were recruited over a two-year period (2015–2017) from a large hospital based pediatric eating disorder service in the Midwest (Minnesota, USA). Participants were recruited as part of a larger effectiveness study examining manualized treatments for patients with DSM-5 eating disorders. The current study examines baseline measures for all patients meeting criteria for ARFID only. Inclusion criteria for participants were: (1) aged 8–18 years; (2) presented for a clinical intake evaluation of eating disorder symptoms; (3) and diagnosed with DSM-5 ARFID based on clinical interviews using the DSM-5 criteria for ARFID with at least two clinicians (a psychiatrist in combination with either a psychologist or clinical social worker), and, when age appropriate, in conjunction with the results from the Eating Disorder Examination (EDE) [15].

Procedures. This study was approved by the appropriate Institutional Review Board (IRB code: 1504-046). Data utilized were part of a larger data collection effort. In addition to the clinical interviews, participants also completed semi-structured interviews (i.e., MINI-Kid, EDE) with a research assistant. Participants were admitted to an inpatient unit if deemed medically unstable by the attending psychiatrist, consistent with appropriate guidelines [16,17]. Once consent and assent were obtained, participants were enrolled into the study.

Group Classifications. *Age*. Given the differences in mental health characteristics by age [18], participants were separated by age and classified as either children (8–11 years old) or adolescents (12–18 years old) for some of our comparisons.

*Weight Status*. Weight status was determined by percent median Body Mass Index (%mBMI). Weight and height were collected on calibrated digital scales and stadiometer in order to calculate a %mBMI. For participants <18 years old, %mBMI was calculated using the median (50th percentile) BMI for age and sex [19]. Weight status was categorized as underweight (%mBMI <90%), or normal weight (%mBMI ≥90%) [20]. Participants were assigned to weight status group based on %mBMI at initial presentation.

Duration of Symptoms. Time of symptom onset was assessed based on self-reported symptoms from parent/guardian and patient at the initial clinical interview. Based on these data and the framework for conceptualizing health conditions [21], participants were grouped into one of the following duration categories: acute (<12 months) or chronic (≥12 months) ARFID symptoms.

DSM-5 Clinical Presentation Coding. Categorization of participants into the DSM-5 designated ARFID presentations was conducted through a chart review by two independent raters (CM and SE). Documents examined included the initial diagnostic assessment completed by both a psychiatrist and a psychologist or clinical social worker. After reviewing each patient chart, the raters independently categorized each case. Given the current research suggesting a dimensional model for ARFID [7], and the likelihood of ARFID patients meeting criteria for more than one presentation, participants were assigned to each presentation for which they met criteria, having the potential to be assigned to up to three ARFID presentations. If either rater disagreed on any dimension, such differences were discussed and reconciled among the two raters. If full consensus was not reached between the raters, disagreements were resolved through a third party rater (HK). Two of the three raters were involved in patient care (SE and HK), but did not code their own patients, and one of the raters was the study coordinator (CM).

Measures. In addition to demographic information about age, gender, race, and ethnicity, a battery of semi-structured interviews and questionnaires were completed.

Diagnostic Interviews. In the absence of a gold-standard diagnostic tool for ARFID, this study used a combination of clinical interviews and measures to confirm ARFID diagnosis. Other eating disorders were ruled out through a combination of clinical interview and the EDE. The *EDE* is a semi-structured interview used to evaluate the frequency and severity of eating disorder symptoms, and was administered to participants aged 12 years and older to discriminate diagnostically between ARFID and more traditional eating disorders, including AN and BN [15].

Medical Presentation. Medical characteristics included orthostatic vital signs: bradycardia (less than 50 beats per minute), and hypotension (measured as a systolic pressure <90). Menstrual cycle status included amenorrhea, which was defined as the absence of menarche age 15 years or older (primary) or the lack of menses for the past 3 months (secondary). Inpatient medical stays were categorized by yes/no, depending on if they were admitted at initial intake. Past history of eating disorder treatment, psychotropic medications, and other medical conditions that could potentially impact weight were also assessed.

Psychological Assessments. *Mini International Neuropsychiatric Interview for Children and Adolescents* (MINI-Kid 5.0) was used to assess comorbid disorders and suicidal and self-harm thoughts and behaviors. Interrater and test–retest reliability for this measure ranges from adequate to perfect (0.64–1.00) [22].

*Child Depression Inventory, Second Edition* (CDI-2) is a 28-item self-report measure that assesses symptoms of depression for children between the ages of 7 and 17 years. The CDI-2 demonstrates good reliability and validity [23].

*Beck Anxiety inventory* (BAI) is a 21-item self-report measure of anxiety [24], with higher scores indicating more severe symptoms of anxiety. It demonstrates good reliability (Cronbach’s Alpha = 92), test–retest reliability, and moderate validity [25]. It has been validated for use in adolescent populations [26]. Participants 12 years and older completed this measure.

*Screen for Child Anxiety Related Emotional Disorders* (SCARED) is a 41 item self and parent report measure that was used to assess anxiety in 8–11-year-olds for this study. This measure demonstrates good reliability and validity [27].

*Rosenberg Self-Esteem Scale* (RSE) is used to assess self-esteem in adult and youth populations, with higher scores indicating higher self-esteem [28]. This measure has been validated in many different languages [29].

*Clinical Impairment Assessment* (CIA) is a 16 item self-report questionnaire [30] that assesses the severity of psychosocial impairment resulting from an eating disorder within the last 28 days. Reliability and validity for this measure support its use in patients with eating disorders [31]. The CIA was completed by adolescents (12 years and older) in our sample.

*Clinical Perfectionism Questionnaire* (CPQ) is a 12-item self-report measure used to assess perfectionism outside of eating and weight concerns [32] that was completed by participants aged 12 years. Higher scores indicate higher clinical perfectionism. It demonstrates good reliability and validity in eating disorder populations [33].

*Child Behavior Checklist* (CBCL) is a parent report scale for children ages 6–17 years old that assesses a broad variety of internalizing and externalizing symptoms. This scale demonstrates strong reliability, as well as convergent and discriminative validity [34].

Statistical Analyses. All demographic, medical, and psychological measures were calculated as means and standard deviations for continuous variables and proportions for categorical variables. Initial analyses for demographic and weight presentation consisted of comparing measures across youth by age, weight status and length of symptoms (e.g., underweight, normal weight, acute symptoms, chronic symptoms). Differences among groups were compared using chi-square test or fisher exact for categorical variables and t-test for continuous variables. Differences between groups were assessed with a *p* < 0.05. All statistical analyses were performed in SAS 9.4 (Cary, NC Carolina).

## 3. Results

*Sample description*. As part of a larger effectiveness study of DSM-5 eating disorder diagnoses, 404 patients were evaluated, of which 338 (84%) met initial eligibility criteria. After in-person evaluation, 266 were verified as eligible, with 102 (39.8%) participants diagnosed with ARFID and included in the present study. Participants were excluded if they: (a) did not meet criteria for an eating disorder, (b) received only inpatient services, (c) met criteria for co-morbid medical disorders known to influence eating or weight (i.e., pregnancy, cancer), (d) presented with a psychotic disorder, (e) were acutely suicidal and/or (f) demonstrated significant substance abuse/dependence. The average age of youth with ARFID was 12.3 years (SD = 2.65) (57.8% between ages of 8–12 years), 82.5% identified as Caucasian, 59.8% were female, 74% (n) = 75) met criteria for at least one other DSM-5 diagnosis, with 57% (n = 58) qualifying for a comorbid anxiety disorder (see Table 1, Table 2 and Table 3). Of note, 16% (n) = 8) of adolescents aged 12–18 years old reported clinically significant levels of anxiety on the BAI, while 29% (n) = 11) of children (aged 8–11 years old) rated clinically significant levels of anxiety on the SCARED. Thirty two percent (n = 16) of adolescents (aged 12–18 years old) rated clinically significant levels of impairment due to disordered eating on the CIA (See Table 1 and Table 2).

*Age comparison*. Results examining psychological variables showed that there were significantly more adolescents (aged 12–18 years) with ARFID and a comorbid Depressive Disorder than their younger counterparts (8–11 years old) (*p* < 0.01, see Table 2). Adolescents presented with significantly lower pulse rates (*p* = 0.05) and higher systolic blood pressure (*p* < 0.01, see Table 3).

*Weight status comparison*. Average %mBMI was 87.1% (normal weight = 36.3%, underweight = 63.7%). By definition, weight measures (e.g., kilograms, BMI, BMI z-score, %mBMI), were significantly different between these two groups (100.4 %mBMI vs. 79.5 %mBMI respectively, *p* < 0.01, see Table 1). Comparisons between normal weight and underweight ARFID youth revealed no significant differences in terms of physiologic parameters (see Table 3). Results examining psychological comorbidities of ARFID indicated that youth with ARFID who also met criteria for a diagnosis of Autism Spectrum Disorder presented with significantly higher weights on average (normal weighted) than their underweight peers (*p* = 0.02). Significant differences were observed across ages for weight and BMI (*p* < 0.01, *p* < 0.01), which is consistent with typical development in youth.

*Symptom duration comparison*. Youth with chronic ARFID symptoms presented with significantly lower BMI z-scores compared to youth with acute ARFID symptoms (chronic = −1.52; acute = −0.86; *p* = 0.03). Males were significantly more likely to experience chronic rather than acute symptoms (*p* = 0.02), while no differences were observed among girls. Those with acute ARFID symptoms endorsed more items on the self-harm/suicidal scale of the MINI kid (*p* = 0.02) compared to those presenting with chronic symptoms.

**Figure 1:**
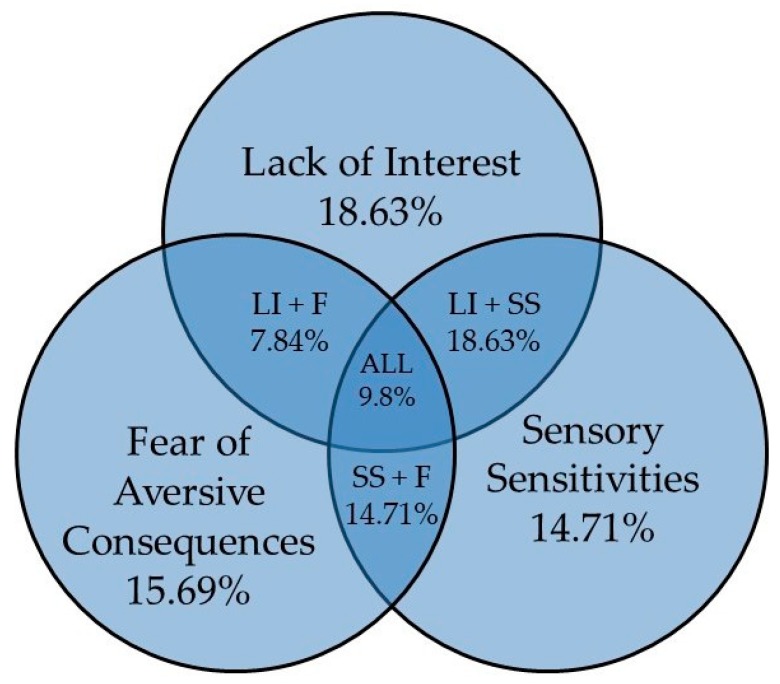
Frequency of Clinical Presentations in ARFID. Note. LI+F = Refers to group that met criteria for Lack of Interest and Fear of Aversive Consequences.LI + SS = Refers to group that met criteria for Lack of Interest and Sensory Sensitivities. SS + F = Refers to group that met criteria for Sensory Sensitivities and Fear of Aversive Consequences. ALL = Refers to group that met criteria for all three clinical presentations.

*ARFID clinical presentation description*. Distribution of DSM-5 clinical presentations within the sample showed that half of patients met criteria for more than one clinical presentation (51%, n = 52); lack of interest (LI = 18.6%, n = 19), sensory sensitivities (SS = 14.7%, n = 15), fear of aversive consequences (F = 16%, n = 16), lack of interest and sensory sensitivities (LI+SS = 18.6%, n = 19), lack of interest and fear of aversive consequences (LI + F = 7.8%, n = 8), sensory sensitivities and fear of aversive consequences (SS + F = 14.1%, n = 15), and lack of interest, sensory sensitivities, and fear of aversive consequences (LI + SS + F = 9.8%, n = 10; see Figure 1).

## 4. Discussion

This exploratory study aimed to examine differences among youth with ARFID by age, weight status, and symptom duration, as well as describe the frequencies of DSM-5 clinical presentations in this heterogeneous treatment-seeking sample at a United States Midwestern (Minnesota, USA) Children’s Hospital. To better understand the differences among individuals with ARFID, our study conducted several exploratory comparisons across age, weight status, and symptom duration.

Our finding that older youth with ARFID were significantly more likely to have a comorbid depressive disorder is consistent with previous studies suggesting that adolescents are more likely to experience a mood disorder than younger children [35]. The findings that adolescents presented with significantly lower pulse rates and higher systolic blood pressure suggest that while there were slight differences in medical presentation by age, these are likely not noteworthy, as they are consistent with age appropriate physiological norms. Likewise, significant differences were noted in average weight (kg) and BMI between children and adolescents, which was expected due to normal growth trajectories.

Comparisons of weight status showed that youth with a comorbid diagnosis of ASD more often presented in the normal weight category. This is meaningful as research is currently concerned about youth with ASD, given that they may be more vulnerable to weight and nutritional difficulties because of selective eating patterns and sensory sensitivities common to the disorder. This finding is interesting because despite eating difficulties, youth with ARFID and ASD may be able to maintain a healthy weight [36,37,38], though more research is needed. While research has recently begun to examine specific associations between youth with ASD and ARFID [39], the present study is among the first to examine the connection between weight status in youth with both ARFID and ASD.

Youth with chronic ARFID symptoms presented with significantly lower weight compared to those with acute symptoms. This is consistent with previous findings that chronicity is typically associated with more negative outcomes in eating disorders [40]. This finding is not surprising clinically, as those with an acute onset of symptoms may present to treatment sooner and with more immediate distress about symptoms than their chronic duration counterparts and therefore not allow weight to reach such severity. That being said, our findings revealed that patients presenting with acute symptom onsets were more likely to endorse suicidal ideation and/or self- harm than their chronic onset peers. This is congruent with previous studies indicating an association between suicidal ideation and/or self-harm and disordered eating [41], and indicates that symptom duration may be an important factor in further understanding this link. These potential associations have important implications for treatment, as youth presenting with acute symptoms of ARFID may need more thorough and timely evaluation of safety concerns even though their weights may not be as low as their chronic counterparts.

Males reported higher rates of chronic symptoms of ARFID than females. Nevertheless, females did not report higher rates of acute symptoms. Given high rates of ARFID within the male population [42], especially when compared to other eating disorder diagnoses, earlier diagnosis and decreasing stigma around treatment may be indicated in order to reduce the chronicity of ARFID symptoms in males.

The absence of significant differences between youth with ARFID and comorbid diagnoses of anxiety disorders by weight status, duration or illness, or age are somewhat unsurprising, given worries and fears tend to be an underlying symptom associated with much of the food avoidance seen in ARFID. In fact, 74% of our sample met criteria for at least one comorbid DSM-5 disorder, which may suggest the benefit of considering a transdiagnostic treatment approach to ARFID that can address the core symptoms of ARFID as well as common comorbidities. This would represent a significant advance in the field of child and adolescent psychology because having a treatment capable of addressing both ARFID and emotional comorbidities would be beneficial for dissemination efforts of evidence based care.

Our results suggest that youth with different ARFID weight histories and clinical presentations look similar across most medical and psychological characteristics, including self-esteem, clinical impairment, perfectionism, and anxiety. This is somewhat surprising considering one might expect to see more difficulties in underweight youth with more chronic symptoms, especially given the previous findings that more significant medical complications and serious psychiatric illness accompany patients who are underweight with chronic symptoms in AN and BN [8]. While there are some similarities between youth with ARFID and other eating disorders, there may also be important differences in the effects of chronicity and weight status that would be helpful to examine further.

This is one of the first studies to report the frequencies of DSM-5 ARFID clinical presentations in all its potential combinations. Half the youth presenting with ARFID in our sample met criteria for more than one of the DSM-5 recommended clinical presentations, and 10% presented with symptoms in all three DSM-5 clinical presentations. This finding suggests that symptom presentations can and often do overlap, and supports Thomas et al.’s [11] call for a dimensional approach to the diagnosis and treatment of these youth.

This study is among the first to conduct a cross-sectional examination of differences within a large sample of youth with ARFID utilizing gold-standard measures. However, it is important to note some limitations to our study. First, post initial assessment chart review was required to code each participant by ARFID presentations. Given the lack of supporting psychometric measures to identify various ARFID presentations at the onset of data collection, clinicians in this study initially made an ARFID diagnosis (yes/no), and did not identify specific presentation types during diagnostic assessments. This may have led to missing presentations if characteristics were not disclosed in patient charts at their initial visit. Research is currently working to rectify this gap in the literature, with new assessments including the Pica, ARFID, and Rumination Disorder Interview (PARDI) and the ARFID module for the EDE in current development [43,44]. Additionally, the modest sample sizes within each different ARFID presentation group prohibited us from further examining clinical and psychological differences between presentations. A future goal might include collaboration between treatment sites to pool data and better describe the psychological and physiological characteristics of the various ARFID symptom presentations. Another potential limitation in our study is that we conducted multiple comparisons which could lead to possible Type I error. However, given the exploratory and descriptive nature of our study, we chose not to control for family-wise error. Finally, the sample for this study was largely ethnically and racially homogenous, hampering our capacity to generalize our findings to more ethnically or racially diverse patient populations.

## 5. Conclusions

Overall, these results are important to the field given that we examined the medical and psychological differences by age, weight status, and duration of symptoms, as well as the frequency of overlapping symptomology among a large sample of ARFID patients. Our findings highlight the heterogeneity within ARFID, including the distinguishable effects of symptom duration on physical and mental health variables, while also showing many important commonalities across age, weight status, and symptom duration. This study supports previous findings that youth with ARFID have high rates of comorbid psychiatric disorders, which indicates that a transdiagnostic approach to its treatment may be a highly beneficial tool for many patients. Lastly, the diversity within ARFID presentations may indicate a dimensional or spectrum approach in conceptualizing the heterogeneity within ARFID.

## Figures and Tables

**Table 1:** Demographic and weight presentation of participants with avoidant/restrictive food intake disorder (ARFID) by age, weight, and acuity (N = 102).

	All N = 102	Normal Weight %mBMI ≥90% N = 37 (36.3%)	Underweight %mBMI <90% N = 65 (63.7%)	*p* ^a^	^c^ Acute Symptom Onset N = 15 (14.7%)	Chronic Symptom Onset N = 86 (84.3%)	*p* ^a^	Children Ages 8–11 N = 43 (42.2%)	Adolescents Ages 12–18 N = 59 (57.8%)	*p* ^a^
**Demographics**										
Age ^d^	12.30 (2.65)	11.95 (2.60)	12.51 (2.68)	0.30	12.53 (2.29)	12.27 (2.73)	0.69			
8–11 years, n (%)	43 (42.2)	18 (48.7)	25 (38.5)	0.32	5 (33.3)	38 (44.2)	0.43	43 (42.2)		
12–18 years, n (%)	59 (57.8)	19 (51.4)	40 (61.5)		10 (66.7)	48 (55.8)			59 (57.8)	
**Gender, n (%)**										
Male	41 (40.2)	16 (43.2)	25 (38.5)	0.64	2 (13.3)	39 (45.4)	0.02	19 (44.2)	22 (37.3)	0.48
Female	61 (59.8)	21 (56.8)	40 (61.5)		13 (86.7)	47 (54.7)		24 (58.8)	37 (62.7)	
**Race, n (%)**										
White/Caucasian	84 (82.4)	33 (89.2)	51 (78.5)	0.17	10 (66.7)	73 (84.9)	0.14 ^b^	36 (83.7)	48 (81.4)	0.76
Black/African American	2 (2.0)	1 (2.7)	1 (1.5)	1.00 ^b^	0 (0)	2 (2.3)	1.00 ^b^	0 (0)	2 (3.4)	0.51 ^b^
Asian	4 (3.9)	0 (0)	4 (6.2)	0.29 ^b^	0 (0)	4 (4.7)	1.00 ^b^	2 (4.7)	2 (3.4)	1.00 ^b^
Ethnicity, n (%)										
Hispanic/Latino	9 (8.8)	3 (8.1)	6 (9.2)	1.00 ^b^	3 (20.0)	6 (7.0)	0.13 ^b^	5 (11.6)	4 (6.8)	0.49 ^b^
**Weight Presentation**										
Kilograms ^d^	37.62 (12.20)	44.25 (14.12)	33.84 (9.09)	<0.01	40.61 (10.83)	37.15 (12.46)	0.28	29.27 (8.12)	43.70 (11.04)	<0.01
BMI ^d^	16.11 (2.70)	18.36 (2.80)	14.84 (1.59)	<0.01	16.98 (2.47)	15.95 (2.74)	0.16	14.92 (2.04)	16.99 (2.81)	<0.01
BMI z-score ^d^	−1.44 (1.40)	−0.10 (0.72)	−2.20 (1.08)	<0.01	−0.86 (0.89)	−1.52 (1.45)	0.03	−1.40 (1.48)	−1.47 (1.36)	0.79
%mBMI ^d^	87.08 (12.90)	100.38 (10.51)	79.51 (6.31)	<0.01	91.29 (11.23)	86.53 (13.05)	0.15	88.16 (12.15)	86.29 (13.46)	0.47

Note. Race excludes patients that declined to respond or selected “other”. ^a^ Analyzed using Pearson’s Chi-square test for categorical variables or *t*-test for continuous variables, unless noted differently. ^b^ Due to small cell count, analyzed using Fischer’s Exact test. ^c^ N = 101 for acute vs. chronic symptom duration analyses. ^d^ Values are means (SD).

**Table 2:** Psychological status of participants with ARFID by age, weight, and acuity (N = 102).

	All N = 102	Normal Weight %mBMI ≥90% N = 37 (36.27%)	Underweight %mBMI <90% N = 65 (63.73%)	*p* ^a^	^g^ Acute Symptom Onset N = 15 (14.71%)	Chronic Symptom Onset N = 86 (84.31%)	*p* ^a^	Children Ages 8–11 N = 43 (42.16%)	Adolescents Ages 12–18 N = 59 (57.84%)	*p* ^a^
**Diagnoses** n (%)										
Psychiatric comorbidity (any DSM-5 diagnosis)	75 (73.53)	26 (70.27)	49 (75.38)	0.57	9 (60.00)	65 (75.58)	0.21 ^b^	30 (69.77)	45 (76.27)	0.46
ADHD	23 (22.55)	7 (18.92)	16 (24.62)	0.51	1 (6.67)	22 (25.58)	0.18 ^b^	13 (30.23)	10 (16.95)	0.11
Autism	6 (5.88)	5 (13.51)	1 (1.54)	0.02	0 (0)	6 (6.98)	0.59 ^b^	1 (2.33)	5 (8.47)	0.40
Depressive Disorder ^c^	19 (18.63)	5 (13.51)	14 (21.54)	0.32	2 (13.33)	17 (19.77)	0.73 ^b^	4 (9.30)	15 (25.42%)	0.04 ^b^
Anxiety Disorder ^d^	58 (56.86)	22 (59.46)	36 (55.38)	0.69	9 (60.00)	48 (55.81)	0.76	23 (53.49%)	35 (59.32%)	0.56
**Parent Report**										
CBCL Score ≥70, n (%)	21 (27.3)	11 (34.4)	10 (22.2)	0.24	1 (8.3)	20 (31.3)	0.16 ^b^	18 (66.67)	38 (76)	0.38
**Self-Report Measures**										
Self-harm/Suicidal ideation ^h^ (MiniKid) ^f,h^	1.91 (0.29)	1.91 (0.30)	1.91 (0.29)	0.86	2.00 (0)	1.89 (0.31)	0.02	1.93 (0.27)	1.90 (0.30)	0.70
CDI Total T Score ≥70, n (%)	7 (9.1)	4 (12.5)	3 (6.7)	0.44 ^b^	0 (0)	7 (10.9)	0.59 ^b^	24 (88.89)	46 (92)	0.69 ^b^
Rosenberg Self-Esteem ^f,h^	21.78 (6.16)	20.91 (6.71)	22.40 (5.73)	0.40	23.58 (6.49)	21.38 (6.11)	0.34	21.07 (6.16)	22.16 (6.19)	0.46
Beck Anxiety Inventory Total Score ^e,h^	10.38 (9.41)	11.65 (9.87)	9.73 (9.25)	0.51	7.44 (9.29)	11.02 (9.42)	0.32			
SCARED Child Total ≥25, n (%) ^i^	11 (29.73)	6 (35.29)	5 (25.00)	0.49						
SCARED Parent Total ≥25, n (%) ^i^	12 (32.43)	6 (35.29)	6 (30.00)	0.73						
BAI Score ≥20, n (%)	8 (16.0)	2 (11.8)	6 (18.2)	0.70 ^b^	1 (11.1)	7 (17.1)	0.66			
CIA Score ≥16, n (%)	16 (32.0)	7 (41.2)	9 (27.3)	0.32	3 (33.3)	13 (31.7)	1.00 ^b^			
Clinical Perfectionism Questionnaire ^e,h^	19.38 (4.95)	19.88 (5.02)	19.12 (4.97)	0.61	18.89 (6.01)	19.49 (4.77)	0.78			

Values are Mean (SD) unless noted differently. ^a^ Analyzed using Pearson’s Chi-square test for categorical variables or *t*-test for continuous variables, unless noted differently. ^b^ Due to small cell count, analyzed using Fischer’s Exact test. ^c^ Patient is considered to have diagnosis of depression if they were diagnosed with Dysthymia, Major Depressive Disorder, Other Specified Depressive Disorder, and/or Unspecified Depression. ^d^ Patient is considered to have an anxiety diagnosis consists if they were diagnosed with OCD, PTSD, Social Anxiety Disorder, Agoraphobia, Generalized Anxiety Disorder, Panic Disorder, Separation Anxiety Disorder, Specific Phobia, Other Specified Anxiety Disorder, and/or Unspecified Anxiety. ^e^ Restricted to n = 50 due to missing data (n = 9) and limited age range (age ≥ 12; n = 59), as younger patients did not complete these measures. ^f^ Removed missing values for internalizing t-score, self-harm, total-t score, and RSE (n = 25) ^g^ N = 101 for acute vs. chronic symptom duration analyses. ^h^ Values are means (SD) ^i^ Restricted to patients 8–11 years old (N = 37) across Weight Status groups. Likewise comparisons were not made across Age groups because only 8–11-year-olds completed the SCARED. Symptom duration was not compared due to low sample size in acute group (n = 3).

**Table 3:** Medical status of participants with ARFID by age, weight, and acuity (N = 102)

	All N = 102	Normal Weight %mBMI ≥90% N = 37 (36.27%)	Underweight %mBMI <90% N = 65 (63.73%)	*p* ^a^	^d^ Acute Symptom Onset N = 15 (14.71%)	Chronic Symptom Onset N = 86 (84.31%)	*p* ^a^	Children Ages 8–11 N = 43 (42.16%)	Adolescents Ages 12–18 N = 59 (57.84%)	*p* ^a^
**Vital Signs**										
Pulse rate ^e^	78.78 (15.10)	76.65 (14.83)	80.00 (15.23)	0.28	76.20 (15.52)	79.42 (15.06)	0.47	82.40 (17.84)	76.15 (12.23)	0.05
Systolic blood pressure ^e^	113.13 (9.54)	115.08 (8.76)	112.02 (9.85)	0.11	113.20 (5.51)	113.17 (10.14)	0.99	109.72 (10.14)	115.61 (8.31)	<0.01
Diastolic blood pressure ^e^	63.32 (7.79)	62.08 (6.86)	64.03 (8.24)	0.20	63.00 (6.68)	63.40 (8.05)	0.84	62.47 (8.69)	63.95 (7.08)	0.36
**Medical Instability**										
Bradycardia, <50 beats per minute, n(%)	4 (3.92)	0 (0)	4 (6.15)	0.29 ^b^	2 (13.33)	2 (2.33)	0.10 ^b^	3 (6.98)	1 (1.69)	0.31 ^b^
Hypotension, systolic pressure <90 mm Hg, n (%)	2 (1.96)	0 (0)	2 (3.08)	0.53 ^b^	1 (6.67)	1 (1.16)	0.28 ^b^	2 (4.65)	0 (0)	0.18 ^b^
Orthostatic Instability, >20 beats per minute, >10 mm Hg, n (%)	56 (54.90)	18 (48.65)	38 (58.46)	0.34	9 (60.00)	46 (53.49)	0.64	21 (48.84)	35 (59.32)	0.29
Admitted/In hospital at presentation, n (%)	14 (13.73)	2 (5.41)	12 (18.56)	0.07	4 (26.67)	10 (11.63)	0.22 ^b^	6 (13.95)	8 (13.56)	0.95
Amenorrhea ^c^, n (%)	6 (9.84)	2 (9.52)	4 (10.0)	1.00 ^b^	3 (23.08)	3 (6.38)	0.11 ^b^	2 (8.33)	4 (10.81)	1.00 ^b^
Prior Eating Disorder Treatment, n (%)	38 (37.25)	15 (40.54)	23 (35.38)	0.60	3 (20.00)	35 (40.70)	0.13	16 (37.21)	22 (37.29)	0.99
Psychotropic medication, n (%)	35 (34.31)	15 (40.54)	20 (30.77)	0.32	4 (26.67)	31 (36.05)	0.48	12 (27.91)	23 (38.98)	0.25
Other medical condition present potentially impacting weight, n (%)	13 (12.75)	5 (13.51)	8 (12.31)	1.00 ^b^	2 (13.33)	11 (12.79)	1.00 ^b^	6 (13.95)	7 (11.86)	0.75

Note. Values are Mean (SD) unless noted differently. Note. Race excludes patients that declined to respond or selected “other”. ^a^ Analyzed using Pearson’s Chi-square test for categorical variables or *t*-test for continuous variables, unless noted differently. ^b^ Due to small cell count, analyzed using Fischer’s Exact test. ^c^ Excludes males (n = 41) ^d^ N = 101 for acute vs. chronic symptom duration analyses. ^e^ Values are means (SD).
